# Prognostic Significance and Gene Expression Profiles of *p53* Mutations in Microsatellite-Stable Stage III Colorectal Adenocarcinomas

**DOI:** 10.1371/journal.pone.0030020

**Published:** 2012-01-19

**Authors:** Venkat R. Katkoori, Chandrakumar Shanmugam, Xu Jia, Swaroop P. Vitta, Meenakshi Sthanam, Tom Callens, Ludwine Messiaen, Dongquan Chen, Bin Zhang, Harvey L. Bumpers, Temesgen Samuel, Upender Manne

**Affiliations:** 1 Department of Pathology, University of Alabama at Birmingham, Birmingham, Alabama, United States of America; 2 Department of Genetics, University of Alabama at Birmingham, Birmingham, Alabama, United States of America; 3 Division of Preventive Medicine, University of Alabama at Birmingham, Birmingham, Alabama, United States of America; 4 Department of Biostatistics, University of Alabama at Birmingham, Birmingham, Alabama, United States of America; 5 Department of Surgery, Morehouse School of Medicine, Atlanta, Georgia, United States of America; 6 Department of Pathology, Tuskegee University, Tuskegee, Alabama, United States of America; 7 UAB Comprehensive Cancer Center, University of Alabama at Birmingham, Birmingham, Alabama, United States of America; Mizoram University, India

## Abstract

Although the prognostic value of *p53* abnormalities in Stage III microsatellite stable (MSS) colorectal cancers (CRCs) is known, the gene expression profiles specific to the *p53* status in the MSS background are not known. Therefore, the current investigation has focused on identification and validation of the gene expression profiles associated with p53 mutant phenotypes in MSS Stage III CRCs. Genomic DNA extracted from 135 formalin-fixed paraffin-embedded tissues, was analyzed for microsatellite instability (MSI) and *p53* mutations. Further, mRNA samples extracted from five p53-mutant and five p53-wild-type MSS-CRC snap-frozen tissues were profiled for differential gene expression by *Affymetrix Human Genome U133 Plus 2.0 arrays.* Differentially expressed genes were further validated by the high-throughput quantitative nuclease protection assay (qNPA), and confirmed by quantitative real-time polymerase chain reaction (qRT-PCR) and by immunohistochemistry (IHC). Survival rates were estimated by Kaplan-Meier and Cox regression analyses. A higher incidence of *p53* mutations was found in MSS (58%) than in MSI (30%) phenotypes. Both univariate (log-rank, P = 0.025) and multivariate (hazard ratio, 2.52; 95% confidence interval, 1.25–5.08) analyses have demonstrated that patients with MSS-p53 mutant phenotypes had poor CRC-specific survival when compared to MSS-*p53* wild-type phenotypes. Gene expression analyses identified 84 differentially expressed genes. Of 49 down-regulated genes, *LPAR6*, *PDLIM3*, and *PLAT*, and, of 35 up-regulated genes, *TRIM29*, *FUT3*, *IQGAP3*, and *SLC6A8* were confirmed by qNPA, qRT-PCR, and IHC platforms. *p53* mutations are associated with poor survival of patients with Stage III MSS CRCs and *p53*-mutant and wild-type phenotypes have distinct gene expression profiles that might be helpful in identifying aggressive subsets.

## Introduction

For patients with colorectal cancers (CRCs), the presence of lymph node metastases is a factor in determining treatment modalities and for predicting clinical outcomes [1]. Since the outcomes for all CRC patients with metastases are not the same [2], there is a need for molecular markers that identify candidates for therapy and predict patient survival. To be clinically useful, such markers should provide information independent of clinicopathologic features.

CRC is a heterogeneous disease that develops through various genetic pathways. The chromosomal instability pathway accounts for 85% of sporadic CRCs [3]. These aggressive tumors are characterized by allelic losses at *17p* and *18q* and by the presence of mutations in common oncogenes and tumor suppressor genes [3], [4]. Chromosomal abnormalities, such as aneuploidy, amplifications, and translocations, are common in familial CRCs [5].

Microsatellite instability (MSI) is associated with the remaining 15% of sporadic CRCs; these tumors are less aggressive and characterized by inefficient DNA mismatch repair [6]. MSI is a characteristic of nearly all cases of hereditary non-polyposis colorectal cancer [7]. Relative to microsatellite-stable (MSS) CRCs, the *APC*, *KRAS*, and *p53* genes are less frequently mutated in sporadic MSI CRCs [8], [9]. Furthermore, for patients with Stage III tumors, *p53* mutations and MSS are associated with poorer prognoses and therapy responses [10], [11], [12]. Thus, these molecular phenotypes are related to the aggressiveness of CRCs. The current investigation is primarily focused on validation of the prognostic value of the p53 status in Stage III MSS CRCs and on identifying and validating the gene expression profiles that are specific to p53 mutant phenotypes in Stage III MSS CRCs. These profiles may lead to identification of therapeutic targets for this subgroup of patients.

## Results

### Characteristics of the study cohort

At the time of surgeries, the mean age of the patients was 65 years (range, 31–94 years). The distribution of Stage III-CRCs in the colorectum was 51, 29, and 20% in the proximal colon, distal colon, and rectum, respectively. There was a preponderance of non-Hispanic Caucasian patients (87 of 135, 64%) and pT3 type of depth of invasion (85 of 135, 63%). At the last follow-up, 28% (34 of 122) of the patients were alive. Those dead because of colorectal neoplasia were 56% (68), and those dead because of other causes was 16% (20).

### Associations between clinicopathological and molecular characteristics

There was a higher frequency of CRCs with the MSS phenotype (112 of 135, 83%) than the MSI-H phenotype (23 of 135, 17%) (Table S1). The incidence of the MSI-H phenotype was higher in the proximal colon (15 of 23, 65%) and in high-grade CRCs (12 of 23, 52%, χ^2^ *P* = 0.03). We also analyzed the distribution of MSI among the three sites (proximal colon, distal colon, and rectum). Consistent with previous studies, there was a higher incidence of the MSI phenotype in proximal tumors (15 of 69, 22%) as compared to distal colon tumors (3 of 38, 8%) and rectal tumors (5 of 27, 19%) (data not shown). The incidence of p53 mutations in Stage III CRCs was 53% (72 of 135). The key observation was that the incidences of *p53* mutations were higher in CRCs with the MSS phenotype (65 of 112, 58%) compared to the MSI-H phenotype (7 of 23, 30%, χ^2^ *P* = 0.02). Relative to wt-*p53*, *p53* mutations were more common in high-grade CRCs (29 of 72, 40%, χ^2^ *P* = 0.04). Among Stage III CRCs, 68% of patients with *p53* mutations died due to CRCs compared to 42% with wt-*p53* (χ^2^ *P* = 0.02). Further analysis of MSS CRCs showed a correlation between *p53* mutations and high-grade disease (23 of 65, 35%, χ^2^ *P* = 0.04) and death due to CRC (χ^2^ *P* = 0.03).

### Gene expression profiles

**Figure 1:**
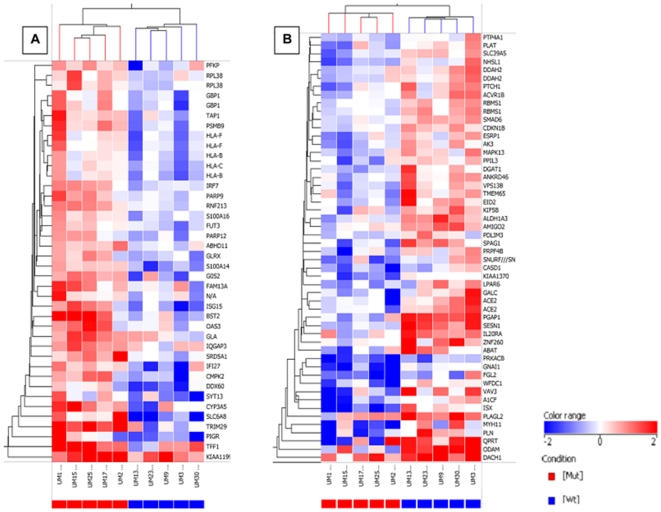
Gene expression profiles of CRCs based on *p53* status. Genes and samples were clustered independently by hierarchical clustering. Rows represent genes, and columns represent samples, which are color-coded by *p53* status (blue and red correspond to wt-*p53* and *p53* mutant, respectively. The color scale is shown at bottom right. Values are expressed as log2-ratios of expression in CRCs with *p53* mutant phenotypes to that in wt-*p53* phenotypes. (A & B) Clusters of 35 and 49 genes showing consistent up-regulation and down-regulation in *p53* mutant phenotypes.

Gene expression analyses identified 84 differentially expressed genes (35 up-regulated and 49 down-regulated) (Figure 1). All of these passed the stringent background filter (intensity *P* = ≤0.001 and intensity ≥3 standard deviation above mean background) and differential expression ratio (χ^2^ *P* = ≤0.05 and fold change ≥2.0). These analyses suggested a transcriptional repression regulated by *p53* mutant phenotypes. In these phenotypes, *GBP1*, *PSMB9*, *BST2*, *FUT3*, *IQGAP3*, *SLC6A8*, *TRIM29*, and *TFF1* genes were up-regulated (Figure 1A) and genes that regulate cell-cycle arrest (*CDKN1B* and *p21^waf-1^*) and tumor growth suppression (*ACVR1B*, *SESN1*, *WFDC1*, *LPAR6*, *PDLIM3*, *PLAT*, and *VAV3*) were down-regulated (Figure 1B).

### Validation of differentially expressed genes

To examine the reliability of microarray data and to validate the expression of differentially expressed genes, all 84 genes were subjected to qNPA. This was performed on 14 wt-*p53* and 14 mutated-*p53* Stage III MSS CRCs matched for patient age, gender, race, tumor location, and tumor grade. For the expression of seven genes, the findings were consistent with those derived with the Affymetrix platform (Table 1). Three genes (*LPAR6*, *PDLIM3 and PLAT*) were down-regulated, and four (*FUT3*, *IQGAP3*, *SLC6A8 and TRIM29*) were up-regulated. qRT-PCR, to measure RNA levels, and IHC analyses, for protein levels of the genes, were then performed for confirmation. The results were similar to those obtained with the qNPA and Affymetrix assay platforms (Table 1).

**Table 1:** Validation of deferentially expressed genes by qNPA and qRT-PCR based on *p53* status in Stage III-MSS CRCs.

				Gene expression					
				AHG^1^		qNPA^2^		qRT-PCR^3^	
Probe ID	Gene Symbol	Ch	Gene name	Fold Change	*P*	Fold Change	*P*	Fold Change	*P*
218589_at	LPAR6	13	Lysophosphatidic acid receptor 6	2.2	0.03	1.4	0.04	1.6	0.02
209621_s_at	PDLIM3	4	PDZ and LIM domain 3	2.2	0.02	1.6	0.04	1.5	0.01
201860_s_at	PLAT	8	Plasminogen activator tissue	2.1	0.04	1.3	0.04	4.3	0.03
214088_s_at	FUT3	19	Fucosyltransferase 3 (Lewis blood group)	2.3	0.005	1.4	0.03	3.5	0.04
229538_s_at	IQGAP3	1	IQ motif containing GTPase activating protein 3	2.1	0.01	1.6	0.009	12.0	0.001
202219_at	SLC6A8	16	Solute carrier family 6 member 8	7.1	0.001	1.6	0.03	1.6	0.02
202504_at	TRIM29	11	Tripartite motif containing 29	7.3	0.001	2.6	0.004	3.1	0.002

Abbreviations: Ch, chromosome number; AHG, Affymetrix human genome plus 2.0 array.

1 Fold change in differential expression of genes by AHG;

2 Fold change by qNPA;

3 Fold change qRT-PCR.

### Immunophenotypic expression analysis

**Figure 2:**
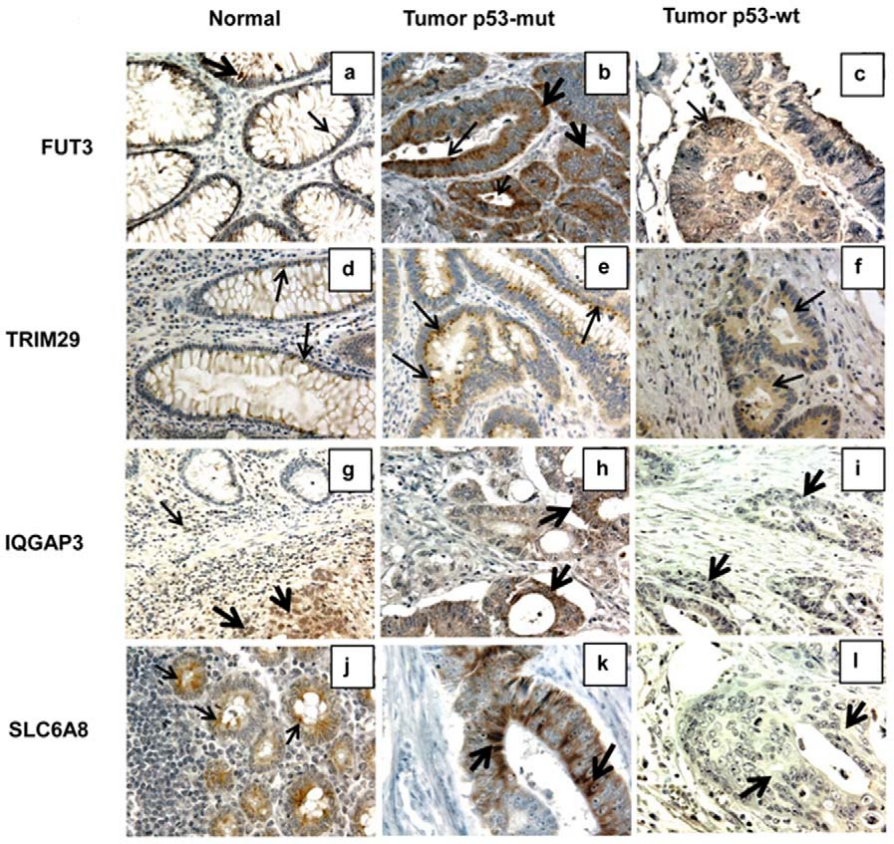
IHC expression patterns of up-regulated genes in *p53* mutant phenotypes of MS CRCs. **a**, normal colonic epithelium (NCE) demonstrating moderate cytoplasmic (thick arrow) and weak membrane (thin arrow) FUT3 staining (400 µm). **b**, CRCs exhibiting strong cytoplasmic (thick arrows) and moderate to weak membrane FUT3 staining (thin arrows) (400 µm). **c**, CRCs with weak cytoplasmic staining (thin arrows) (400 µm). **d**, NCE demonstrating weak cytoplasmic TRIM29 staining with a focal punctuate pattern (thin arrows) (400 µm). **e**, CRCs exhibiting moderate to strong cytoplasmic TRIM29 staining with a punctate pattern on the luminal aspect (thin arrows) (600 µm). **f**, CRCs with weak cytoplasmic TRIM29 staining (thin arrows) (400 µm). **g**, NCE demonstrating weak cytoplasmic to complete lack of IQGAP3 staining. [Note: Lymphocytes in the stroma show moderate cytoplasmic staining (thin arrows); the adjacent tumor demonstrates moderate cytoplasmic and nuclear immunostaining (thick arrows) (400 µm)]. **h**, CRCs exhibiting strong cytoplasmic IQGAP3 staining (thick arrows) (400 µm). **i**, CRCs with lack of staining for IQGAP3 (thick arrows) (400 µm). **j**, NCE demonstrating moderate cytoplasmic staining of SLC6A8 staining (thin arrows) (600 µm). **k**, CRCs exhibiting strong cytoplasmic SLC6A8 staining with luminal accentuation (thick arrows) (600 µm). **l**, CRCs negative for SLC6A8 staining (thick arrows) (600 µm).

**Figure 3:**
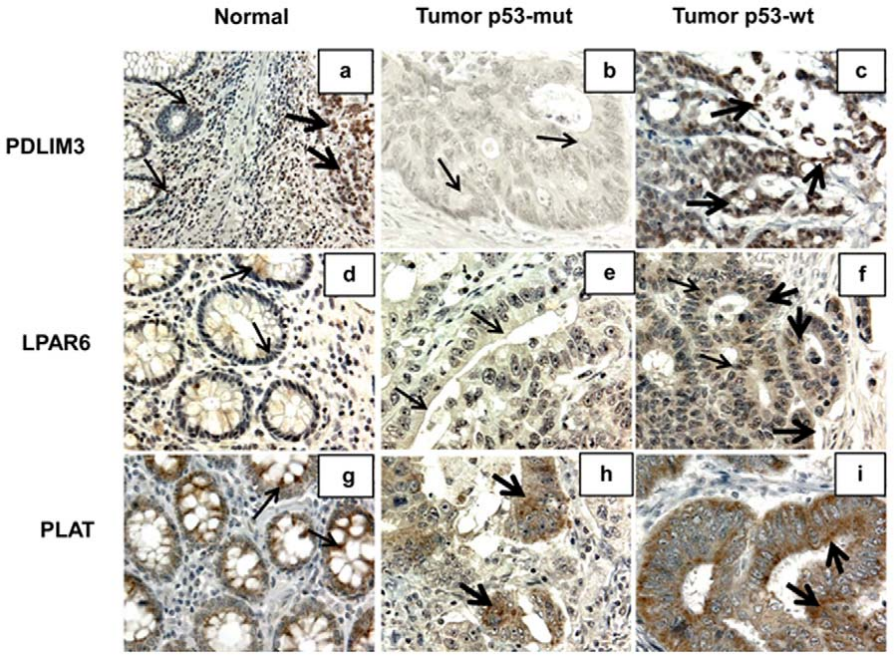
IHC expression patterns of down-regulated genes in *p53* mutant phenotypes of MS CRCs. **a**, NCE demonstrating moderate nuclear and weak cytoplasmic PDLIM3 staining [Note: Lymphocytes in the stroma and the normal epithelial cells show moderate nuclear staining (thin arrows); the adjacent tumor demonstrates strong nuclear and moderate cytoplasmic immunostaining (thick arrows) (400 µm)]. **b**, CRCs exhibiting lack of PDLIM3 immunostaining (thin arrows) (600 µm). **c**, CRCs with strong nuclear and weak cytoplasmic PDLIM3 staining (thick arrows) (600 µm). **d**, NCE demonstrating weak cytoplasmic LPAR6 staining (thin arrows) (600 µm). **e**, CRCs exhibiting focal weak cytoplasmic LPAR6 staining (thin arrows) (600 µm). **f**, CRCs with weak to moderate cytoplasmic (thick arrows) and focal nuclear LPAR6 staining (thin arrows) (600 µm). **g**, NCE demonstrating moderate to strong cytoplasmic PLAT staining (thin arrows) (600 µm). **h**, CRCs exhibiting focal moderate cytoplasmic PLAT staining (thick arrows) (600 µm). **i**, CRCs with moderate to strong staining for PLAT (thick arrows) (600 µm).

There were differences in expression of these markers between CRCs with and without *p53* mutations as well as tumors versus normal colonic epithelium (NCE). There was moderate-to-strong staining for FUT3, IQGAP3, SLC6A8 and TRIM29 in samples with *p53* mutations compared to those with wt-*p53* (Figure 2). In contrast, staining for LPAR6, PDLIM3, and PLAT was moderate in CRCs with wt-*p53* as compared to those with *p53* mutations (Figure 3). NCE demonstrated moderate cytoplasmic and weak membrane FUT3 staining, whereas CRCs exhibited strong to weak cytoplasmic and moderate to weak membrane FUT3 staining. TRIM29 exhibited weak cytoplasmic staining in the NCE and moderate to strong cytoplasmic staining with a punctate pattern on the luminal surface. In the NCE, IQGAP3 demonstrated weak to absence of staining in the cytoplasm; lymphocytes showed moderate cytoplasmic staining. CRCs exhibited strong cytoplasmic or lack of staining for IQGAP3. SLC6A8 staining was moderate in the cytoplasm and, in CRCs, there was strong cytoplasmic staining with luminal accentuation. Moderate nuclear and weak cytoplasmic PDLIM3 staining was seen in NCE. Lymphocytes in the stroma showed moderate nuclear staining and served as an internal control. For PDLIM3, CRCs exhibited either lack of staining or strong nuclear and weak cytoplasmic staining. There was weak staining for LPAR6 in the cytoplasm of NCE. CRCs demonstrated weak to moderate cytoplasmic and focal nuclear staining. NCE and CRCs demonstrated moderate to strong cytoplasmic staining for PLAT.

### Survival analyses

**Figure 4:**
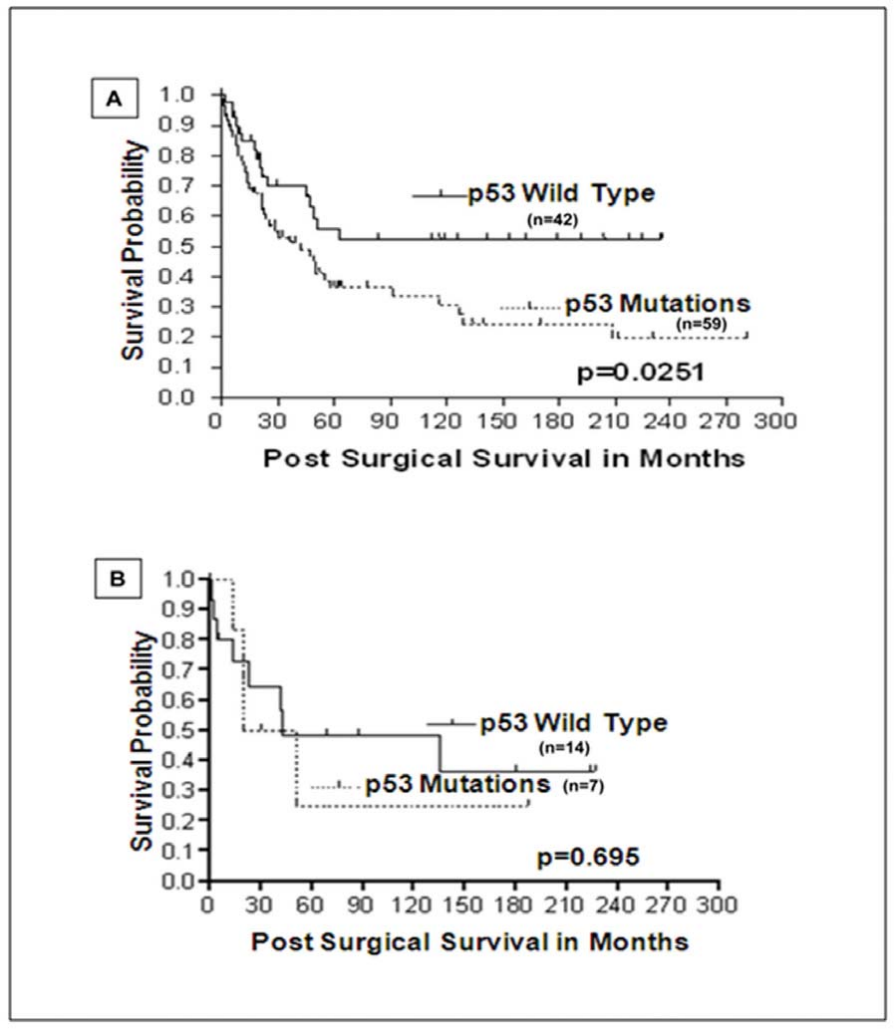
The prognostic significance of *p53* mutations in survival of Stage III CRC patients with MSS or MSI-H phenotypes (Kaplan-Meier survival curves). *p53* mutations were associated with worse patient survival (log-rank, *P* = 0.025) (**A**) than wt-*p53* in the subset of MSS phenotype, but not in the subset of MSI-H phenotype (log-rank, *P* = 0.695) (**B**).

Univariate Kaplan-Meier survival analyses for the MSS phenotype group demonstrated that CRCs with *p53* mutations (n = 59) were significantly associated with shorter disease-specific survival relative to those with wt-*p53* (n = 42) (log rank, *P* = 0.025) (Figure 4A). For patients with the MSI-H phenotype, CRCs with *p53* mutations did not demonstrate such a difference (log-rank, *P* = 0.695) (Figure 4B).

Evaluation of the prognostic significance of *p53* mutations on CRC-specific survival of MSS Stage III CRCs confirmed the independent effect of *p53* mutations on CRC-specific survival (Table 2). Overall, patients with *p53* mutations were 2.52 times (hazard ratio [HR = 2.52]; 95% confidence interval [95% CI], 1.25–5.08) more likely to die of CRC compared to those with wt-*p53* (log-rank, *P* = 0.01). Age (≥65), distal colon site, and pN2 (greater number of nodes with metastases) were independent prognostic indicators of MSS Stage III CRC patients (Table 2).

**Table 2:** Cox regression analysis to determine prognostic significance of *p53* mutations.

Prognostic variables	Indicator of poor prognosis	Hazard ratio^1^(95% confidence intervals)	*P*
*p53* status			
Mutated vs. wt	Mutated	2.52 (1.25–5.08)	0.01
Tumor grade			
High vs. low	High grade	1.11 (0.59–2.09)	0.74
Tumor location			
Proximal vs. distal colon	Proximal colon	0.38 (0.18–0.81)	0.01
Proximal colon vs. rectum	Proximal colon	0.71 (0.35–1.46)	0.35
Distal colon vs. rectum	Distal colon	0.25 (0.11–0.58)	0.001
Distal vs. proximal colon	Proximal colon	0.38 (0.18–0.81)	0.01
Tumor size			
>5 cm vs. ≤5 cm	>5 cm	0.77 (0.42–1.41)	0.4
Age			
≥65 vs. <65	≥65	2.30 (1.23–4.33)	0.01
Gender			
Male vs. female	Male	0.84 (0.45–1.58)	0.59
Race			
African Americans vs. Caucasians	African Americans	0.98 (0.49–1.96)	0.95
pT status			
pT1 vs. pT2, pT3, pT4	pT2,pT3, pT4	1.23 (0.74–2.03)	0.43
pN status			
pN1vs. pN2	pN2	1.84 (1.09–3.09)	0.02

1 Adjusted for the *p53* mutations, age, gender, tumor location, tumor grade, tumor size, tumor depth of invasion, nodal involvement, and adjuvant therapy.

The median *p*-value for each variable obtained from the bootstrapping of the Cox regression models was selected as an estimate of the statistical significance. The median *p*-value for the *p53* mutations in MSS CRCs was 0.005 (relative to wt). Since the median *p*-values (0.01) were consistent with the final model (Table 2), the results were robust.

## Discussion

The prognostic value of *p53* mutations in MSS phenotypes of Stage III CRCs was assessed, and other molecular markers associated with *p53* mutant phenotypes were identified. The prevalence of *p53* mutations was higher in CRCs with the MSS phenotype (59%) as compared to the MSI-H phenotype (30%). Both univariate and multivariate survival analyses revealed that *p53* mutations in Stage III-MSS-CRCs were associated with shorter cancer-specific survival relative to those with wt-*p53*. After rigorous validation on four platforms, a molecular signature associated with *p53* mutant phenotypes in this subset of CRCs was identified.

The differentially expressed genes are involved in remodeling of the extracellular matrix, adhesion, cytoskeleton plasticity, and signal transduction. The *p53* mutant signature was characterized by transcriptional repression, which is distinct from the expression associated with wt-*p53*. Thus, metastasis mediated by *p53* mutations is a selective process with a specific molecular signature of Stage III-MSS CRCs.

In about 50% of human cancers, including CRCs, the *p53* gene is mutated. The gain of oncogenicity or loss of tumor suppressor function of *p53* due to its inactivation through missense mutations contributes to tumor aggressiveness and results in poor patient survival [13], [14], [15], [16], [17], [18], [19]. *p53* mutations have been considered as metastatic signatures in CRCs [20]. The mutations that affect structural or functional domains and those in evolutionary conserved regions are associated with aggressive tumors [21] and chemo-resistance [22]. *p53* mutations within certain domains [L2 and L3 loops, and the Loop Sheet Helix (LSH) motif] and mutations in evolutionary non-conserved region of the *p53 gene* [23] are associated with aggressive phenotypes [24]. These observations, in conjunction with our previous findings [25], suggest a function for the p53 protein in metastasis due to inactivation and indicate that patients with CRCs exhibiting *p53* mutations are at risk for aggressive progression and early death. The present study demonstrates an association between *p53* mutations and poor survival in Stage III-MSS-CRCs. Genomic instability, common in CRCs [26], correlates with *p53* mutations [27]. Thus, for some Stage III-MSS-CRCs, genomic instability associated with *p53* mutations is the cause of developing a risk phenotype and for poor survival.

Previous studies have demonstrated that sporadic colon cancers with the MSI phenotype are less aggressive, because these tumors commonly show a lower frequency of p53 mutations [28], as observed in the current study. This relationship is consistent with the concept that most CRCs develop either along the chromosomal instability pathway associated with *TP53* mutations and MSS tumors or the aberrant mismatch repair pathway associated with wild-type p53 and MSI tumors [8], [29], [30]. This association may also explain why tumors with MSI seem to have less aggressiveness as compared to those with a proficient mismatch repair system. In the current investigation, there was no significant association between p53 status and poor patient survival for Stage III CRCs with the MSI phenotype, suggesting different effects of p53 mutations in Stage III CRCs with MSS and MSI phenotypes.

Microarray analyses, based on the *p53* status in Stage III-MSS CRCs, identified 84 genes differentially expressed in tumors with *p53* mutant and wt-*p53* phenotypes. In tumors of the *p53* mutant phenotype, 35 genes were up-regulated and 49 down-regulated, suggesting that transcriptional repression of genes is important for developing risk of aggressive cancer and for poor survival manifested by *p53* mutations. The repression affects known tumor or metastasis suppressor genes, such as *TRIM 29*, *ACVR1B*, and *LPAR6* [31], and the sestrins, *SESN1* and *SESN3* [32]; however, in our analyses, ACVR1B, LPAR6, and sestrins were not validated by the qNPA method. Thus, the results support the concept that silencing of genes is essential for tumor progression [33]. One explanation for this is that the inactivation of *p53* by mutation results in the overexpression of transcriptional repressors, which suppress other genes, including those suppressing metastasis.

Gene microarray is an excellent approach to evaluate the expression profiles of thousands of genes; however, it has several limitations related to accuracy and reproducibility [34], [35]. For a differentially expressed gene to serve as a biomarker with high accuracy, verification of analysis of gene expression by multiple methods is required [36]. Inaccuracies in identifying the ‘true’ expression of potentially useful molecular markers may be the basis for the lack of translation of the findings obtained from studies involving gene microarrays. Thus, to validate results obtained from these microarrays, the genes selected in the present investigation were subjected to the quantitative nuclease protection assay (qNPA) and qRT-PCR. The qNPA assay validated only seven of the differentially expressed genes identified by Affymetrix microarrays. These seven genes were further validated by qRT-PCR and IHC. There are associations between dysregulation of all seven genes and cancer development [37], [38], [39], [40], [41].

Although little is known about the properties of *TRIM29*, a member of the tripartite motif (TRIM) family, three proteins of this family (TRIM19, TRIM24 and TRIM27) are involved in cellular growth or development [42] and become oncogenic as a result of chromosomal translocations [43], suggesting their involvement in tumor progression. Increased expression of *TRIM29* is associated with tumor differentiation, tumor growth, tumor invasion, and lymph node metastasis [44]. In the present study, TRIM29 was over-expressed in *p53* mutant phenotypes, supporting its function in p53 dependent-pathways that lead to aggressive behavior of Stage III-MSS CRCs. Further mechanistic studies are needed to clarify the function of TRIM29 in CRC progression.

IQGAP family proteins modulate cytoskeletal architecture and cell adhesion [45], [46], and they may be involved in metastasis of CRCs, lung cancers, and cholangiocarcinomas [47]. *IQGAP3*, which encodes a putative 180-kDa protein with RasGAP, IQG1, CH, and COG5022 domains, regulates cell proliferation through the Ras/ERK signaling cascade [48]. Our findings show up-regulation of IQGAP3 in MSS and *p53* mutant phenotypes and indicate that, for Stage III-MSS CRCs, the underlying molecular mechanism for IQGAP3 is different in *p53* mutant and wt-*p53* phenotypes. Also, the results indicate that IQGAP3 is involved in metastasis. Although it might be useful for predicting CRC progression, its function and prognostic capacity remain to be investigated.

It is important to compare the gene expression profiles of MSS Stage III CRCs with and without *p53* mutations to understand the molecular mechanisms involved in their progression and to identify aggressive subsets. The present results, demonstrating that *p53* mutations are associated with a poor prognosis for Stage III microsatellite-stable CRCs, may lead to development of individualized therapies for Stage III-MSS CRCs. Furthermore, robust validations of the expression profiles, including those for *TRIM29* and *IQGAP3*, may allow identification of novel therapeutic targets.

## Materials and Methods

### Patient population

The Institutional Review Board of the University of Alabama at Birmingham (UAB) approved these studies and we obtained informed written consent from all study participants. The UAB Bioethics Committee reviewed the proposed effort.

From 1986 through 2004, there were 303 patients with Stage III CRCs at the UAB Hospital. Use of these patients maximized post-surgery follow-up. The following were excluded from the study: those who died within a week of surgery (n = 20); those with surgical margin-involvement (n = 16), unspecified tumor location (n = 15), multiple primaries within the colorectum (n = 14), multiple malignancies (n = 32), unknown tumor grade (n = 5), those with a family history of hereditary non-polyposis colorectal cancer (n = 6), familial adenomatous polyposis coli (n = 4), a personal history of CRC (n = 11), or inflammatory bowel disease (n = 9). Also excluded, were patients who received pre-surgical chemo- or radiation therapy (n = 19).

Formalin-fixed, paraffin-embedded (FFPE) tissue blocks or snap-frozen CRC tissues were obtained from the Anatomic Pathology Division. These CRCs and corresponding normal tissues (colonic tissues collected 8 cm from CRCs) were analyzed. Suitable tissue specimens were unavailable for 17 patients. Thus, there were 135 patients for analysis.

### Treatment

Of these 135 patients, 51 had received adjuvant chemotherapy. Fifteen patients received 5-fluorouracil (5-FU) alone, 9 received 5-FU plus levamisole (LV), 8 received 5-FU plus leucovorin (LC), 14 received 5-FU/leucovorin/oxaliplatin, 3 received 5-FU plus doxorubicin, 1 received 5-FU/1-(2-chloroethyl)-3-cyclohexyl-1-nitrosourea), and 1 received 5-FU/LV/LC. The remaining 84 patients received only surgery. None of these patients received neoadjuvant therapy. The number treated with adjuvant therapy was low because the Food and Drug Administration approved the usage of 5-FU-based adjuvant chemotherapy for advanced stages of CRCs only in the early 1990's.

### Pathological features

Two pathologists (CKS & WEG) individually reviewed slides stained with hematoxylin and eosin (H and E) for the degree of histologic differentiation and re-graded lesions as well-, moderate-, poor-, or undifferentiated. Cases with disagreement were resolved by reevaluating the slides to reach a consensus. Well and moderately differentiated tumors were pooled into a low-grade group and poor and undifferentiated tumors into a high-grade group [49].

Pathologic staging conformed to the criteria of the American Joint Commission on Cancer [50]. The International Classification of Diseases for Oncology codes were used to specify the anatomic locations of tumors [51]. The anatomic sites were the proximal colon, distal colon, and rectum. Of the tumor dimensions, the largest was used for statistical purposes. Before the tissues were analyzed, a section was cut from each block and stained with H and E to permit macro-dissection to separate tumor from uninvolved tissue.

### Patient demographics and follow-up information

Patient demographics and clinical and follow-up information were retrieved retrospectively from medical records, physician charts, pathology reports, and from the UAB Tumor Registry. Patients were followed by their physicians or by the Registry until their death or the date of the last documented contact within the study timeframe. This information was validated against the state death registry. The mean follow-up period, which ended in December 2010, was 10.9 years (<1 to <22 years). The laboratory investigators (VRK, XJ, & CS) were blinded to the outcome information until completion of the assays.

### Microsatellite instability analysis

DNA extraction from FFPE archival tissues was performed following a modified deparaffinization protocol [25]. Five polymorphic microsatellite markers (BAT25, BAT26, D2S123, D5S346, and D17S250) from the Bethesda consensus panel [52]. were amplified by PCR using marker-specific, fluorescence-labeled microsatellite primers (Applied Biosystems Inc, Foster City, CA) [53]. The reaction mixture (25 µl) consisted of 10 ng of genomic DNA, 10× PCR buffer, 1.5 mM MgCl_2_, 50 mM dNTPs, 10 pmol of each primer, and 0.3 units of platinum Taq DNA polymerase (Invitrogen, Carlsbad, CA). The thermal profile was as follows: after initial denaturation at 94°C for 5 min, amplification was accomplished for 36 cycles, 30 sec at 94°C, 30 sec at 55°C, and 1 min at 70°C. The final extension step was at 70°C for 7 min. The 6FAM-, HEX, and NED-labeled PCR products (1 µl each) was added to the mixture of 13 µl deionized formamide and 1 µl of gene scan 500 TAMRA. This preparation was denatured at 88°C for 4 min, followed by chilling on ice for 2 min and centrifugation for 15 sec. The denatured samples were analyzed with an ABI 3100 genetic analyzer (Applied Biosystems) using the Performance Optimized Polymer4 gel in a 47-cm×50-µm capillary. The data, collected automatically, were analyzed by Genotyper 2.1 software (Applied Biosystem). MSI was determined by the presence of one or more additional peaks in tumor samples as compared to corresponding normal samples.

### p53 mutational analysis

The status of the *p53* gene was assessed by PCR and direct sequencing of exons 4 through 9, by use of exon-specific primers [25]. The purified PCR product was sequenced on an ABI 3100 sequencer (Applied Biosystems). Compilation and sequence analysis was performed with LASERGENE (DNA STAR Inc, Madison, WI) software.

### Microarray for expression profiling

Gene expression analyses of CRC samples were conducted using Affymetrix Human GeneChip U133 plus 2.0 arrays to determine the profiles based on the *p53* status in Stage III MSS CRCs. The gene expression studies were performed on surgically resected specimens of five wt-*p53* and five mutant *p53* Stage III MSS CRCs. These mutations, localized at codons 126, 135, 183, 248, and 276, are inactivating mutations [16]. For gene expression analyses, 5 µg of total RNA, extracted from snap-frozen specimens, was used. The quantity and quality of the RNA was determined by the RNA nanochip on an Agilent BioAnalyzer. The transcriptional activity of genes was determined by hybridizing fluorescently labeled, first-strand cDNAs corresponding to p53 mutant versus wt-p53 categories of MSS Stage III CRCs, to a microarray as described earlier [54].

Statistical and bioinformatics analyses of the GeneChip data were conducted using GeneSpring (Agilent) and Partek (St Louis, MO) software. The raw GeneChip files were uploaded, background was subtracted, variance was stabilized, and normalized by GC-RMA [55]. The expression levels in normal (benign epithelial) tissues were used to calculate the intensity ratio or fold change of CRCs with MSS *p53* mutant and MSS wt-*p53* separately, and to compare these two groups for identification of differentially expressed genes. Multiple hypotheses testing, such as Benjamini-Hochberg false discovery rate *P*-value correction, was performed for all comparisons [55]. If there were no significant differences in gene expression levels, however, we performed only the t-test. *P*-values and fold changes were used to identify candidate genes.

To generate a heat-map, raw data were quantile-normalized, log2-transformed, and intensity-filtered before being subjected to an unpaired t-test. The selected gene lists were clustered by hierarchical methods and visualized as normalized, log2-transformed intensities using GeneSpring (Agilent, CA, US).

The gene expression data is ‘minimum information about a microarray experiment’ (MIAME) compliant and the raw data has been deposited with the gene expression omnibus (GEO) of NCBI, and the accession number is GSE27157. The following link has been created to allow review of record GSE27157: http://www.ncbi.nlm.nih.gov/geo/query/acc.cgi?token=hnaflkocasqwmju&acc=GSE27157.

### Quantitative nuclease protection assay (qNPA)

To confirm the expression levels of key genes identified by the Affymetrix GeneArrays (Figure 1), qNPA was performed on RNA [56] extracted from FFPE CRC sections [57], and the processed samples were transferred to programmed (linker-modified) ArrayPlates (High Throughput Genomics, Inc, Tucson, AZ) [56], [57]. The ArrayPlates that received the chemiluminescent peroxidase substrate were viewed from the bottom with an OMIX HD imager. The digital images of ArrayPlates were analyzed by ArrayPlate Fit (v.3.31a) software. The resulting data were analyzed by ArrayPlate Crunch software to normalize signals with β-actin and to calculate gene expression levels.

### Quantitative real-time-PCR

RNA (1 µg) from the tissues was reverse transcribed by PCR as performed in SYBR green reagent supermix (Bio-Rad laboratories, Hercules, CA) consisting of a gene-specific, real-time (RT) primer set [58]. PCR reactions were performed using an i-Cycler RT-PCR system (Bio-Rad). PCR products were subjected to melting curve analysis to exclude non-specific amplification. All PCR reactions were performed in sets of four. The means of the specific gene mRNA and *β*-actin mRNA copy numbers were calculated for each patient separately, and ratios were generated.

### Immunohistochemistry

Tissue sections (5-µm) were cut from paraffin blocks representative of normal and tumor tissues of each case and were mounted on Superfrost/Plus (Fisher Scientific, Pittsburg, PA). IHC was performed as described earlier [59], [60]. In brief, heat-induced epitope (antigen) retrieval was performed on the sections for 10 min with citrate buffer (0.01 M, pH 6) for LPAR6 and PDLIM3; and EDTA buffer (0.01 M, pH 9) for PLAT, FUT3, IQGAP3, SLC6A8, and TRIM29. Tissue sections were then incubated with polyclonal antibodies for LPAR6, PDLIM3, PLAT, IQGAP3, and SLC6A8 (Santa Cruz Biotech Inc, CA) for TRIM29 (Abnova, Walnut, CA), or with monoclonal antibody (121 SLE mouse monoclonal) for FUT3 (Santa Cruz Biotech Inc). Sections to which the antibody was not applied served as negative controls. Secondary detection was accomplished using a multi-species detection system (Signet Lab Inc., Dedham, MA). A diaminobenzidine tetrachloride super-sensitive substrate kit (BioGenex, San Ramon, CA) was used to visualize antibody-antigen complexes. Then each section was counterstained with hematoxylin, dehydrated with graded alcohols, and soaked in xylene before cover-slipping.

### Statistical Methods

#### Sample size and power calculations

The sample size and power analysis were estimated based on a prior Stage III CRC study [61]. In that study (n = 70), p53 mutations increased the risk of death by 2.39 times in patients with Stage III CRC. Based on these results [61], there was enough power to detect a hazard ratio ≥2.39. Therefore, the sample size (n = 135) was sufficient to identify a significant prognostic value for p53.

#### Statistical analyses

Deaths due to CRC were the outcomes (events) of interest. Patients who died within one month after surgery were excluded. Since 13 of 135 patients (11 with the MSS and 2 with the MSI phenotype) were lost to follow-up, survival analyses were performed on the remaining 122 (101 with MSS and 21 with MSI) based on their *p53* mutational status. Chi-square tests were used to compare baseline characteristics in each group [62]. These analyses were also used to examine univariate associations with covariates and potential confounders. The baseline characteristics included demographic (age, gender and race/ethnicity) and pathologic variables (tumor location, depth of invasion, nodal involvement, tumor grade, and tumor size). All analyses were performed with SAS statistical software, version 9.2 [63]. Survival analysis was used to model time from date of surgery until death due to CRC. Those patients who died of any cause other than CRC and those who were alive at the end of the study were censored. A log-rank test and Kaplan-Meier survival curves [64] were used to compare the survival in each group. The type I error rate of each test was controlled at <0.05.

In addition to the primary analysis determining the effect of the *p53* mutations, secondary analyses were performed to consider covariates known to be confounders or independent risk factors for death. These included age; gender; tumor location, depth of invasion, and nodal involvement; tumor grade and size; and adjuvant chemotherapy. For these analyses, Cox regression models [65] were used within each molecular group, with a final model including those covariates for which *P*<0.05.

The bootstrap method was used to demonstrate the robustness of the results. By re-sampling the rows of the data matrix with replacement, separate datasets (200) were constructed [66]. The final models were fit to each of the datasets. The median *P*-values for each variable were selected as estimates of the statistical significance.

## Supporting Information

Table S1 Table S1 describes association between clinicopathological and molecular characteristics based on p53 or MSI status of Stage III CRCs.
(DOCX)
