# Supplementary material for: Prognostic Significance and Gene Expression Profiles of p53 Mutations in Microsatellite-Stable Stage III Colorectal Adenocarcinomas
Source: PLoS One. 2012 Jan 19;7(1):e30020. doi: 10.1371/journal.pone.0030020 (PMC3261849; doi:10.1371/journal.pone.0030020)
Supplement: Table S1 — describes association between clinicopathological and molecular characteristics based on p53 or MSI status of Stage III CRCs. (DOCX) [file pone.0030020.s001.docx]

| **Table S1**. Association between clinicopathological and molecular characteristics based on p53 or MSI status of Stage III CRCs | | | | | | | | | | |
| --- | --- | --- | --- | --- | --- | --- | --- | --- | --- | --- |
|  | | | | | | | | | | |
| ***P53* Status**  **MSI Status** **MSS CRCs** | | | | | | | | | | |
| **Variable** | **Wt-p53** | **Mut-p53** | ***P*** | **MSS** | **MSI** | ***P*** | **Wt-p53** | **Mut-P53** | ***P*** |  |
|  | **N=63**  **(%)** | **N=72**  **(%)** |  | **N=112**  **(%)** | **N=23 (%)** |  | **N=47**  **(%)** | **N=65**  **(%)** |  |  |
| Age group (years |  |  |  |  |  |  |  |  |  |  |
| <65 | 36 (57) | 30 (42) | 0.07 | 54 (48) | 12 (52) | 0.73 | 26 (58) | 27 (42) | 0.12 |  |
| ≥65 | 27 (43%) | 42 (58) |  | 58 (52) | 11 (48) |  | 21 (42) | 38 (58) |  |  |
| Gender |  |  |  |  |  |  |  |  |  |  |
| Female | 32 (51) | 32 (44) | 0.46 | 55 (49) | 9 (39) | 0.38 | 24 (51) | 31 (48) | 0.64 |  |
| Male | 41 (49) | 40 (56) |  | 57 (51) | 14 (61) |  | 22 (49) | 34 (52) |  |  |
| Race/Ethnicity |  |  |  |  |  |  |  |  |  |  |
| African American | 23 (37) | 25 (35) | 0.83 | 38 (34) | 10 (43) | 0.38 | 16 (21) | 22 (34) | 0.92 |  |
| Non-Hispanic Caucasians | 40 (63) | 47 (65) |  | 74 (66) | 13 (57) |  | 31 (79) | 43 (66) |  |  |
| Tumor location^1^ |  |  |  |  |  |  |  |  |  |  |
| Proximal colon | 31 (50) | 38 (53) | 0.81 | 54 (49) | 15 (65) | 0.19 | 19 (40) | 35 (54) | 0.34 |  |
| Distal colon | 17 (27) | 21 (29) |  | 35 (31) | 3 (13) |  | 16 (36) | 19 (29) |  |  |
| Rectum | 14 (23) | 13 (18) |  | 22 (20) | 5 (22) |  | 11 (24) | 11 (17) |  |  |
| Depth of invasion |  |  |  |  |  |  |  |  |  |  |
| pT1 | 1 (1) | 1 (2) | 0.71 | 2 (2) | 0 (0) | 0.93 | 0 (0) | 1 (1) | 0.52 |  |
| pT2 | 10 (16) | 7 (9) |  | 14 (13) | 3 (13) |  | 8 (17) | 7 (11) |  |  |
| pT3 | 37 (59) | 48 (67) |  | 70 (63) | 15 (65) |  | 27 (57) | 43 (66) |  |  |
| pT4 | 15 (24) | 16 (22) |  | 26 (23) | 5 (22) |  | 12 (26) | 14 (22) |  |  |
| Nodal involvement |  |  |  |  |  |  |  |  |  |  |
| pN1 | 36 (57) | 43 (60) | 0.76 | 66 (59) | 13 (56) | 0.83 | 30 (64) | 39 (60) | 0.68 |  |
| pN2 | 27 (43) | 29 (40) |  | 46 (41) | 10 (44) |  | 17 (36) | 26 (40) |  |  |
| Tumor grade |  |  |  |  |  |  |  |  |  |  |
| Low | 48 (76) | 43 (60) | 0.04 | 80 (71 ) | 11 (48) | 0.03 | 38 (82) | 42 (65) | 0.04 |  |
| High | 15 (24) | 29 (40) |  | 32 (29) | 12 (52) |  | 9 (17) | 23 (35) |  |  |
| Tumor size^1^ |  |  |  |  |  |  |  |  |  |  |
| ≤5 | 29 (50) | 28 (41) | 0.32 | 46 (44) | 11 (50) | 0.62 | 25 (60) | 34 (55) | 0.63 |  |
| >5 | 29 (50) | 40 (59) |  | 58 (56) | 11 (50) |  | 17 (40) | 28 (45) |  |  |
| *p53* status |  |  |  |  |  |  |  |  |  |  |
| Wild Type | - | - | - | 47 (42) | 16 (70) | 0.02 | - | - |  |  |
| Mutations | - | - |  | 65 (58) | 7 (30) |  | - | - |  |  |
| MSI status |  |  |  |  |  |  |  |  |  |  |
| MSS | 47 (75) | 65 (90) | 0.02 | - | - | - | - | - | - |  |
| MSI-H | 16 (25) | 7 (10) |  | - | - |  | - | - |  |  |
| Vital status at the end of follow-up^2^ |  |  |  |  |  |  |  |  |  |  |
| Alive | 21 (37) | 13 (20) | 0.02 | 27 (27) | 7 (33) | 0.60 | 15 (37) | 11 (19) | 0.03 |  |
| Died due to CRC | 24 (42) | 44 (68) |  | 56 (55) | 12 (57) |  | 16 (39) | 39 (66) |  |  |
| Died due to other than CRC | 12 (21) | 8 (12) |  | 18 (18) | 2 (10) |  | 10 (24) | 9 (15) |  |  |
| Abbreviations: MSS, microsatellite stable; MSI-H, microsatellite instability high; CRC, colorectal adenocarcinoma. ^1^Information on tumor location for one case and tumor size for 9 cases was not available. ^2^Information as described in Materials and Methods; 13 patients were lost to follow-up | | | | | | | | | | |
